# Adhesion of Different Resin Cements to Zirconia: Effect of Incremental versus Bulk Build Up, Use of Mould and Ageing †

**DOI:** 10.3390/ma15062186

**Published:** 2022-03-16

**Authors:** Nicolas Müller, Nadin Al-Haj Husain, Liang Chen, Mutlu Özcan

**Affiliations:** 1 Center of Dental Medicine, Division of Dental Biomaterials, Clinic for Reconstructive Dentistry, University of Zurich, Plattenstrasse 22, 8032 Zurich, Switzerland; nicolas.mueller@zzm.uzh.ch (N.M.); nadin.al-haj-husain@zmk.unibe.ch (N.A.-H.H.); 2 Department of Reconstructive Dentistry and Gerodontology, School of Dental Medicine, University of Bern, 3010 Bern, Switzerland; 3 BISCO Inc., Schaumburg, IL 1100, USA; lchen@bisco.com

**Keywords:** adhesion, adhesive cementation, ageing, bond strength, macroshear, test method, zirconium dioxide

## Abstract

Bonding to zirconia presents a great challenge, as the clinical guidelines for predictable adhesion are not sufficiently validated. The aim of this study was to assess the influence of various bonding methodologies of various resin cements on zirconia, using different aging protocols. Manufactured zirconia specimens (N = 300 and n = 20 per group) were randomly assigned to three luting protocols: 1—in mould incremental build up; 2—in mould incremental build up with mould removal; 3—in mould non-incremental bulk build up. Five dual, photo- and chemical-cure resin cements were used, namely, Variolink Esthetic (Ivoclar), Tetric (Ivoclar), Panavia (Kuraray), TheraCem (Bisco), and RelyX UniCem (3M ESPE), and were applied on primed zirconia using photopolymerization protocols. Thereafter, the specimens were subjected to the following three ageing methods: 1—dry; 2—thermocycling (×5000; 5–55 °C); 3—3–6 months of water storage. Using a universal testing machine, the specimens were loaded under shear, at 1 mm/min crosshead speed. An analysis of the data was performed using three-way ANOVA and the Bonferroni method. The moulding type, ageing and luting cement significantly affected the results (*p* < 0.05). Among all the protocols under dry conditions, TheraCem (16 ± 3; 11 ± 1; 16 ± 3) showed the best bond strength, while, after thermocycling, TheraCem (7 ± 2) and Tetric (7 ± 2) performed the best with Protocol 1. In Protocol 2, RelyX (7 ± 3) presented the highest result, followed by TheraCem (5 ± 3) and Tetric (5 ± 1) (*p* < 0.05). Using Protocol 3, RelyX (10 ± 6) showed the highest result, followed by TheraCem (7 ± 2) and Panavia21 (7 ± 2) (*p* < 0.05). Six months after water storage, TheraCem presented the highest result (10 ± 2) in Protocol 1, while, in Protocols 2 and 3, Tetric (10 ± 2; 15 ± 5) presented the highest result, followed by TheraCem (6 ± 2; 8 ± 3). Adhesion tests using the incremental or bulk method, using moulds, showed the highest results, but removing the mould, and the subsequent ageing, caused a decrease in the adhesion of the resin cements tested on zirconia, probably due to water absorption, with the exclusion of Tetric.

## 1. Introduction

The demand for full-ceramic restorations has grown in the field of dentistry, resulting in a decline in the use of conventional metallic materials. Full-ceramic restorations are highly aesthetic with their tooth-coloured appearance, and they meet the demands of dentists and patients. Given their superior biocompatibility [1], outstanding mechanical properties [2], and excellent performance [3], zirconia-based ceramics are attracting widespread popularity in daily clinical practice. This goes hand in hand with the widespread implementation of computer-aided design/computer-aided manufacturing (CAD/CAM) technologies in the field of dentistry. In most cases, pre-sintered ceramic blocks are used to mill the restorations, which are only sintered to full density afterwards [4]. Thus, zirconia, as a dental material, allows the fabrication of a great number of clinical applications, including the following: endodontic posts, implant abutments, orthodontic brackets, single crowns, and fixed dental prostheses (FDPs) [5].

Several situations in the clinic, e.g., when there is not enough retention given by a short or tapered tooth structure, mandate adhesive bonding by using conventional adhesive or self-adhesive resin cement. Firstly, it has been proven that the fracture resistance and longevity of ceramic restorations are improved by sealing the internal surface flaws. Secondly, it offers the advantage of improving marginal adaptation and preventing microleakage at the restoration’s margin [6]. Additionally, slightly fractured dentures and crowns demand ceramic repair.

The crucial prerequisite for adequate adhesion is to generate a stable composite–zirconia bond by the combination of physical microretention and chemical surface activation [7,8]. However, the composition of high-strength zirconia ceramics is different, as they lack a silica phase with glassy components that can be etched, resulting in chemical inertness with a surface that is low in reactivity [9]. In the case of acid-etchable silica-based ceramics (>15 wt.% glass), etching the surface with hydrofluoric acid (5–9.6%) is a common method for bonding resin-based materials [10]; however, it is not effective in creating a microretentive surface on zirconia ceramics, and even leads to degradation in the mechanical properties [11,12,13].

To tackle the clinical difficulty of adhering resin cements to zirconia, a plethora of surface conditioning methods has been introduced recently [7,8,9]. A routine practice employed by dental practitioners involves airborne particle abrasion of the zirconia ceramic surface with silica-coated aluminium oxide (Al_2_O_3_) particles to achieve micromechanical interlocking. Moreover, the embedded silica-coated particles render the surface more chemically reactive, so that it is prepared for silanization via silane coupling agents. Silanes, as bifunctional molecules, guarantee adhesion between the ceramic (inorganic) and the resin (organic) bonding agent, by forming siloxane bonds [14] with the ceramic (inorganic) part and copolymerizing with the resinous phase. Additionally, the cement wettability and surface energy of ceramics are improved [15]. This process is referred to as tribo-chemical coating [9,16,17,18].

Surface grinding with a fine diamond bur has also been advocated, in order to form small irregularities on the substrate, allowing the resin composite to flow into the substrate [19]. Despite its advantages, there is a concern that roughening procedures by air abrasion or grinding may cause flaws in the zirconia. On the one hand, roughening and air abrasion induce a tetragonal to monoclinic phase transformation, resulting in higher reliability and flexural strength, while, on the other hand, superficial defects may decrease the mechanical properties and lead to failure. These two effects are contradictory and need to be in balance, and several factors (i.e., grit size, pressure, and distance of the nozzle) may play an important role [20,21,22,23]. The available literature seems to be conflicting, although air abrasion with 30 mm Al_2_O_3_ particles with a silica-coating yields the best results [24,25].

Recently, non-invasive alternatives, such as nano-structured alumina coating [26] or special zirconia primers, based on organophosphate and carboxylic acid monomers [27], have been propagated to eliminate aggressive conditioning approaches. In addition, the introduction of primers containing 10-methacryloyloxydecyl dihydrogen phosphate (MDP), acting as universal primers through a similar hydroxylation-driven chemistry, has received much attention [28]. Bifunctional phosphate monomers can also be part of the resin cement. Besides MDP-based cements, several other adhesive cements, with different compositions, have been tested. More recent evidence highlights the fact that the way of conditioning the surface, in combination with resin cement selection, significantly influences the bond strength to zirconia [8].

Much of the recent work in this field has focused on different surface conditioning protocols and cement types. However, to the best of the authors’ knowledge, no study has investigated the influence of the incremental and bulk application of resin cement to zirconia. Such a methodological factor may affect the adhesion results dramatically. The cement is enclosed under the intaglio surface, and is simulated in this in vitro using a mould. Therefore, the objective of this in vitro study was to examine the effect of different luting techniques (incremental, bulk build up, and the use of mould) on the adhesive potential of resin cements to zirconia with and without ageing. The null hypotheses were that the luting method, aging and type of cement would not have a significant influence on the adhesion of the cements to zirconia.

## 2. Materials and Methods

**Figure 1:**
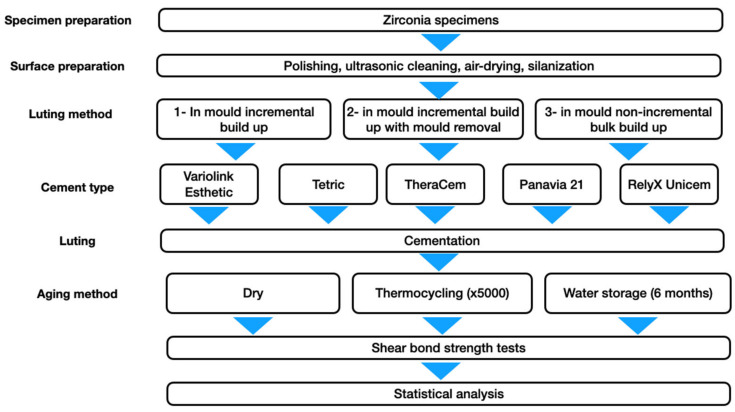
Flowchart outlining experimental arrangement and alignment of groups.

The different materials, brands and chemical structures used in this study are listed in Table 1. Arrangement of laboratory groups based on the luting methods, cement types and ageing procedures is displayed in Figure 1. Power analysis was performed with statistical software for the given specimen size (SPSS software, V.20 and G*Power, IBM, Armonk, NY, USA).

### 2.1. Specimen Preparation

Zirconia specimens (N = 300, n = 20) were cut into slices (12 × 12 × 2 mm^3^) from pre-sintered Y-TZP blocks (IPS e.max ZirCAD, Ivoclar Vivadent, Schaan, Lichtenstein) under water coolant using a precision cutting machine (Struers, Accustom-50, Struers A/S, Ballerup, Denmark) according to ISO 6872-2008 [29]. They were embedded in epoxy resin (Condular AG, Wager, Switzerland) exposing one side of the disk for bonding. To remove any irregularities, the disks were ground finished by the use of silicon carbide papers in the order of 600, 8000, and 1200 grit (WS flex 18 C, Hermes, Virginia Beach, VA, USA) under continuous water cooling (Streuers, Willich, Germany) until a homogenous surface was obtained. The surfaces were ultrasonically cleaned in isopropyl alcohol (Vitasonic II, Vita, Bad Säckingen, Germany) for 5 min and allowed to dry at room temperature.

### 2.2. Luting Methods

**Figure 2:**
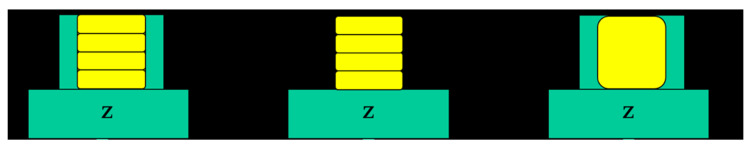
Illustration of the three application modes: mould filled incrementally, mould removed after incremental build up, and mould filled in bulk. z: Zirconia surface.

The specimens were randomly distributed to one of the following luting protocols (Figure 2):

Protocol 1: in mould incremental build up;
Protocol 2: in mould incremental build up with subsequent mould removal;
Protocol 3: in mould non-incremental bulk build up.

First, a thin layer of silane coupling agent (Monobond Plus, Ivoclar Vivadent, Schaan, Liechtenstein) was added through a disposable, clean brush each time. It was allowed to set for 60 s and any remaining excess was removed with pure air. Translucent polyethylene moulds (inner diameter: 3 mm; height: 4 mm) were fixed with a holder on the conditioned substrate. For each luting method, five different cements were used, namely, Variolink Esthetic (Ivoclar); Tetric (Ivoclar); Panavia 21 (Kuraray, Osaka, Japan); TheraCem (Bisco, Schaumburg, IL, USA); RelyX UniCem (3M ESPE, St. Paul, MN, USA).

The self-adhesive RelyX UniCem and TheraCem cements are dual-cure resin cements. Tetric and Variolink Esthetic are light and Panavia 21 is a chemical-cure resin cement.

The cements were handled according to the manufacturers’ recommendations at room temperature and inserted into the polyethylene, according to Protocols 1, 2 and 3. In Protocols 1 and 2, incremental build-up was employed with each increment having a height of 1 mm, resulting in four layers.

Each side of the specimens was photo-polymerised for 40 s (Bluephase, light intensity: 1000 mW/cm^2^), and a glycerine gel (Oxguard II, Kuraray) was placed on the ceramic–composite interface of the layer applied last for 3 min, to prevent oxygen inhibition, and rinsed off afterwards.

The bonded specimens were then randomly assigned to one of the following 3 ageing subgroups: 1: dry; 2: thermocycling for 5000 cycles between 5 and 55 °C with dwelling time at each temperature of 30 s, resulting in 60 cycles per hour (Willytech, Gräfelfing, Germany); 3: water storage for 6 months in distilled water. The dry testing group was included to evaluate the immediate shrinkage effect on the first increment. Dry specimens were stored for 24 h in dark at 37 °C in an incubator (Binder, Fishersci, Switzerland).

### 2.3. Macroshear Test

The specimens were placed in the jig of the universal testing machine (Z010, Zwick Roell, Ulm, Germany) and shear force was exerted as near as possible to the adhesive bond using a cylindrically formed testing rod at a crosshead speed of 0.5 mm/min until failure occurred, according to ISO/TS 11405 [30]. The stress–strain curve was traced with the corresponding software program (TestXpert V11.02 Master, Zwick Roell, Ulm, Germany). Bond strength S (MPa) was calculated using the formula S = A:L, where L is the force at failure (N) and A is the adhesive area (=7.065 mm^2^).

### 2.4. Statistical Analysis

Statistical analysis was performed using SPSS software (IBM, Armonk, NY, USA). Descriptive statistics, including minimum, maximum, mean values and corresponding 95% confidence intervals, were computed. Three-way analysis of variance (ANOVA) was used for comparisons and pairwise comparisons were performed by the Bonferroni method. *p* values < 0.05 were considered to be statistically significant in all tests.

## 3. Results

The type of moulding, the ageing and the chosen luting cement significantly affected the bond strength results (*p* < 0.05), as illustrated in Table 2.

The aging method under dry conditions presented in all the protocols applies the significantly highest bond strength values when the dual-cure resin cement TheraCem (16 ± 3; 11 ± 1; 16 ± 3) was used, only non-significantly different from Tetric (*p* > 0.05).

**Figure 3:**
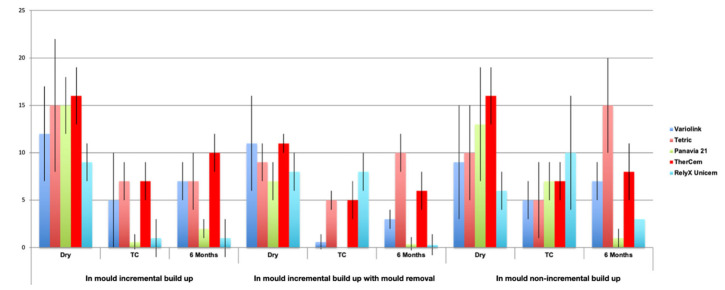
Mean and standard deviation values (MPa) for the 5 cements (Variolink, Tetric, Panavia 21, TheraCem and RelyX Unicem) for the 3 protocols and ageing methods (dry, thermocycling and 6 months of water storage).

When thermocycling was applied, the dual-cure resin cement TheraCem (7 ± 2) and the light-curing cement Tetric (7 ± 2) presented the significantly highest results when using Protocol 1 (*p* > 0.05), while RelyX (7 ± 3) delivered the best bond strength, followed by TheraCem (5 ± 3) and Tetric (5 ± 1) when using Protocol 2. In Protocol 3, RelyX (10 ± 6) presented the significantly highest results, followed by Panavia (7 ± 2) and TheraCem (7 ± 2) (*p* < 0.05), as shown in Figure 3.

**Figure 4:**
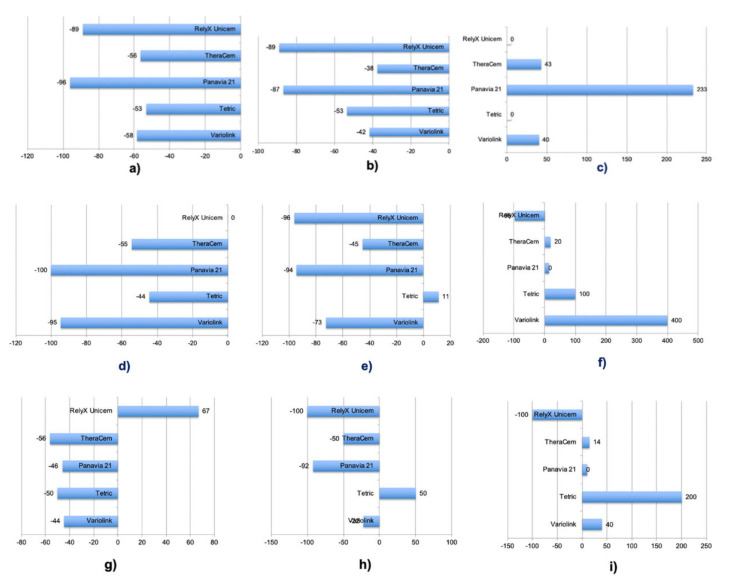
(**a**–**i**) In mould incremental build up (Protocol 1): (**a**) Δdry—thermocycling, (**b**) Δdry—6 months of water storage, (**c**) Δthermocycling—6 months of water storage. (**d**–**f**) In mould incremental build up with subsequent mould removal (Protocol 2): (**d**) Δdry—thermocycling, (**e**) Δdry—6 months of water storage, (**f**) Δthermocycling—6 months of water storage. (**g**–**i**) In mould non-incremental bulk build up: (**g**) Δdry—thermocycling, (**h**) Δdry—6 months of water storage, (**i**) Δthermocycling—6 months of water storage.

After 6 months of water storage, in Protocol 1, TheraCem showed the significantly highest results (10 ± 2), and in Protocols 2 and 3, Tetric (10 ± 2; 15 ± 5) showed the significantly highest result, followed by TheraCem (6 ± 2; 8 ± 3). When incremental build up using mould was practiced (Protocol 1), the smallest decrease after 6 months of water storage ageing was observed with TheraCem (*p* < 0.05). After incremental build up using mould and subsequent removal of the mould (Protocol 2), the smallest decrease after 6 months of water storage ageing was observed with TheraCem (*p* < 0.05), and a significant increase was even observed with Tetric (11%) (*p* < 0.05). When non-incremental build up (bulk) (Protocol 3) using mould was performed, after 6 months of water storage, an increase was also observed with Tetric (50%) (*p* < 0.05), while the smallest decrease was observed with Variolink (22%), yet this decrease was not significant (*p* > 0.05) (Figure 4). All failures occurred at the adhesion interface and were considered to be adhesive failures.

## 4. Discussion

This study was conducted in order to investigate the effect of different luting techniques (incremental, bulk build up, and use of mould) on the adhesion of resin cements to zirconia, with and without ageing. Based on the results of this study, since the moulding type, luting cement and ageing significantly affected the results, the null hypotheses could be rejected.

As the established bonds between an adherent and a substrate are submitted to a combination of both shear and tensile torque forces during chewing in service, different testing methodologies—namely, macrotensile, microtensile, macroshear and microshear tests—have been proposed to measure the bond strength values between dental ceramics and resin-based materials. Regardless of the procedure, it is mandatory that the most stress is forced on the bonding interface zone [31], which should range from 3 mm^2^ to 1 mm^2^ in macro and micro-testing arrangements, respectively [32]. The universal way of ranking the adhesive performance of adhesive resin cements is performed using moulds, and by filling them incrementally, depending on the height of the mould. Although it may not completely reflect the thin cement film under restoration, the first layer of increment is, in fact, decisive on the adhesive strength of the cement. There are attempts to bond ceramic to ceramic with a thin film of cement, which may be more clinically relevant. However, surface conditioning method onto both ceramic surfaces interferes with the outcome, and, thus, may not be completely clinically representative. Therefore, considering all systematic reviews and meta-analyses in the field, we chose to use the mould technique. Several adhesion studies in dentistry, with a similar methodology, have stated that some bond strength tests do not adequately stress the interfacial area [33,34]. Since micro-test methods cover a diminished bonded field with more homogenous stress distribution, they are likely to deliver significantly higher bond strength data than macro-test methods, eventually leading to false ranking of the examined materials [35]. Moreover, micro-tensile tests are also prone to pre-test failures during the cutting of bonded interfaces in high-strength ceramics [33]. In contrast, it is assumed that microshear tests are a more reliable alternative for testing bond strength in small areas. However, the viscosity of the luting cements tested would not show the necessary rheology to apply them in small tubes. Thus, in this study, a macroshear test was used to evaluate the bond strength values, which is also the most frequently applied test method as a simple and repeatable testing option [8]. According to an ISO standard, a widely accepted threshold for adequate shear bond strength is 5 MPa [36], and shear bond strength under 13 MPa should be considered critically [8].

Since there is some speculation that air abrasion methods may create possible damage to ZrO_2_ ceramics, some companies have started to advertise special zirconate primers, based on organophosphate/carboxylic acid monomers, as a different option to the established mechanical pre-treatments [37]. These zirconate primers might bond with the hydroxyl groups on the zirconium dioxide surface. Enhanced bonding to resin has been reported, but a significant decrease was observed after thermocycling [38]. Bifunctional phosphate monomers, containing 10-methacryloyloxydecyl phosphate monomers (MDP), adhere to oxides of ZrO_2_ ceramics by generating a direct bond between the ceramic surface and the resin [39]. Manufacturers advise that these MDP-based products be used without any physical or chemical pre-treatment, even if it is controversially considered that the addition of mechanical surface conditioning and/or silane coupling agents may deliver improved adhesion [40,41]. Thus, in this study, all the specimen surfaces were only pre-treated with Monobond Plus, a primer containing MDP and silane bi-functional silane molecules, to evaluate the true bonding potential of the tested cements, without any physical surface pre-treatment. Several studies indicated favourable adhesion of MDP-based cements, known for their chemical interaction potential, and the results in this study are in accordance with those of previous studies [7,8]. In dry conditions, TheraCem produced the highest bond strength values in all the luting protocols (1–3), as an MDP-based cement. The chemical-cure resin cement Panavia 21, one of the most frequently used resin cements in dentistry, showed the second highest bond strength values in Protocols 1 and 3. However, when the mould was removed subsequently after build up in Protocol 2, Panavia 21 performed worse, probably due to water uptake. In general, incremental or bulk application of the resin cement using the mould (Protocols 1 and 3) delivered significantly (*p* < 0.05) higher bond strength values than when the mould was removed (Protocol 2).

In direct composite restoration, incremental build up has been generally recommended, in order to reduce polymerisation shrinkage and its associated stress [42]. However, bulk-fill composite materials have gained more attention lately in the dental community. Interestingly, in this study, when the cements were bulk filled in Protocol 3, the dual-cure resin cement TheraCem and the chemical-cure Panavia 21 performed similarly, in terms of bond strength values, compared to when they were built up incrementally in Protocol 1. This finding implies that bulk filling using mould might be a suitable and simple solution. This option prevents the replacement of the whole ceramic reconstruction, and the lifetime can be prolonged in a more conservative way.

To estimate the long-term behaviour in clinics, the bonded joints are exposed to different ageing conditions. The dry specimen testing represents the early failures immediately after cementation, when the cement is not exposed to aging of any water uptake. While thermocycling represents in vitro hydrothermal ageing, water storage mimics ageing by water uptake and, consequently, hydrolytic degradation. Thermocycling may simulate the worst-case ageing scenario, with its temperature changes leading to repetitive contraction–expansion stresses at the cut surface. The 6 months of water storage serves to expose the cement to water sorption, excluding possible temperature changes [43]. At least 5000 cycles are advised for metal–resin adhesion tests, according to the ISO norm [36]. More studies are necessary to carry out some standardization of the ageing protocol. While most of the studies on ceramic–resin adhesion involved different thermocycling times, the common consensus was that thermocycling decreased the bond strength when zirconia did not receive any mechanical pre-treatment [7]. After ×5000 thermocycling, the self-adhesive dual-cure resin cement RelyX presented the smallest decrease in Protocol 2 (*p* > 0.05) and Protocol 3 (*p* < 0.05). The superior results of RelyX are in line with the observations of a previous study [14]. In Protocol 1, the self-adhesive dual-cure resin cement TheraCem performed the best, both after ×5000 thermocycling and after 6 months of water storage.

The performance of MDP-based cements after ageing has been discussed controversially. On the one hand, hydrolytic degradation weakens the chemical hydroxyl bindings formed between the MDP monomer and the zirconia ceramic surface [44,45]. On the other hand, when zirconia was pre-treated with a tribo-chemical coating, and an MDP-containing primer/cement was used, hardly any ageing effect was found [7,8,46]. However, silane molecules react under chemical reactions, forming hydroxyl bonds, which also takes place under ageing conditions. Thus, a more hydrolytically and/or thermally stable siloxane bond between the resin-based adhesive and the silica-coated ceramic surface is established. In short, the advantages of using an MDP monomer support the lower efficiency of the surface modification in dry conditions, and the silica-coated surface benefits from the hydrothermal stability of the MDP monomer after ageing [47].

Interestingly, after 6 months of water storage, the bond strength values for the light-curing resin cement Tetric did not decrease, but increased significantly, in Protocol 3 (*p* < 0.05), and not significantly in Protocol 2 (*p* > 0.05). Generally, adhesion with cements containing UDMA, TEGDMA or Bis-GMA is impaired after ageing [48], and this phenomenon was also demonstrated by Variolink in this study. Depending on the composition, the bond becomes weaker, not only due to water uptake, but as a result of increased hydrolysis. However, longer water storage periods need to be administered to investigate this effect.

Today, clinicians are still constantly faced with the dilemma of whether to follow the cement manufacturers’ instructions during the cementing of zirconia, or to modify the instructions by following a different surface conditioning protocol. There is still scarce knowledge as to whether air abrasion has a harmful effect on the fatigue strength of zirconia. Furthermore, from a clinical point of view, sufficient bond strength is difficult to define. Considering the high values obtained in this study, it might not be necessary to aim for even higher bond strength values, as the retention decrease in zirconia reconstructions is reported to be a seldom event, according to clinical studies for crowns and FDPs [49]. However, for resin-bonded FDPs, retention solely relies on adhesion, and the information obtained in this study may be useful for clinical protocols.

The results of this study can apply to all kinds of adhesion protocols. The clinical survival of resin-bonded FDPs benefits more from the results of these studies, as they lack mechanical retention compared to full-coverage FDPs. Yet, the methodology employed during bonding resin-based materials may also affect the outcomes. In this study, the materials were either not exposed to water, or stored only in water, whereas, in the oral environment, adhesive interfaces are exposed to saliva and other acidic agents, which were simulated in this study and can, therefore, be considered as one of the limitations of the study. It also has to be noted that the cement thickness between the restoration surface and enamel/dentin is certainly much thinner than each increment applied in the mould in this study. This could also be considered as a limitation of not only this study, but all in vitro adhesion studies, which could be addressed in future studies with a different mould design.

Nonetheless, there is room for continued development, and more studies are necessary to better elucidate the effect of ageing on both methacrylate and MDP-based resin cements.

## 5. Conclusions

From this in vitro study, the following could be concluded:

1. The adhesion of the tested resin cements to zirconia was influenced by the cement type, luting protocol and ageing.
2. In dry conditions, the MDP-based cement TheraCem performed the best in all the adhesion protocols.
3. After ageing, a reduction in bond strength was observed among most cement types, as a result of hydrolytic degradation. As an exception, Tetric showed an increase in bond strength, possibly due to further polymerization and a higher degree of conversion.
4. Considering the higher bond strength values overall, MDP-based cements may adhere to a zirconium surface more reliably by using a universal primer based on MDP and MPS, and this can be suggested as an option for bonding to zirconia without air abrasion.

## Figures and Tables

**Table 1:** The main chemical compositions and brands of the cements and substances used for the experiments. BPEDMA: bisphenol-A-polyethoxy dimethacrylate, DMA: aliphatic dimethacrylate, HEMA: 2-hydroxyethyl methacrylate, MDP: 10-methacryloyloxy-decyl-dihydrogenphosphate, F3Yb: ytterbium trifluoride, UDMA: urethane dimethacrylate.

Material	Chemical Composition	Manufacturer
Monobond Plus	Alcohol solution of silane methacrylate, phosphoric acid methacrylate and sulphide methacrylate	Ivoclar Vivadent, Schaan, Liechtenstein
Oxyguard II	Glycerol 50–70 wt.%, polyethylene glycol, catalysts, accelerators, dyes	Kuraray, Osaka, Japan
RelyX Unicem (REL)	Phosphoric acid methacrylates, dimethacrylates, silanated fillers, inorganic fillers (72 wt.%), initiators, stabilizers, rheologic additives	3M ESPE, St. Paul, MN, USA
Tetric (TET)	F3Yb, Bis-GMA, urethandimethacrylate, triethylenglycoldimetharcylate	Ivoclar Vivadent, Schaan, Lichtenstein
TheraCem (THC)	Paste A: Portland Cement, Yb with barium glass, F3Yb, BisGMA Paste B: MDP, HEMA, tert-butyl perbenzoate	BISCO Inc., Schaumburg, IL, USA
Panavia 21 (PAN)	Paste A: BPEDMA, MDP, DMA, silanated silica filler silanated colloidal silica Paste B: DMA, pigments, accelerators	Kuraray, Osaka, Japan
Variolink esthetic (VAR)	Monomer matrix: UDMA, methacrylates Inorganic filler: YbF3, spheroid mixed oxide, inorganic Fillers (38 wt.%: particle size: 0.04–0.2 mm, mean: 0.1 mm)	Ivoclar Vivadent, Schaan, Lichtenstein

**Table 2:** Effect of cement in each moulding method, with *p* values showing significant differences between the mean bond strength values (MPa) of the resin-based cement to the zirconia surface in Protocols 1–3. For groups’ abbreviations, see Table 1.

Protocol 1	P1-PAN		P1-THC		P1-VAR		P1-TET		P1-REL	
P1-PAN			0.000		0.005		0.000		n.s.	
P1-THC	0.000				0.000		n.s.		0.000	
P1-VAR	0.005		0.000				0.001		0.000	
P1-TET	0.000		n.s.		0.001				0.000	
P1-REL	n.s.		0.000		0.000		0.000			
**Protocol 2**	**P2-PAN**		**P2-THC**		**P2-VAR**		**P2-TET**		**P2-REL**	
P2-PAN			0.000		0.000		0.000		n.s.	
	
P2-THC	0.000				0.001		n.s.		0.000	
	
P2-VAR	0.005		0.001				0.000		n.s.	
	
P2-TET	0.000		n.s.		0.000				0.000	
	
P2-REL	n.s.		0.000		n.s.		0.000			
	
**Protocol 3**	**P3-PAN**		**P3-THC**		**P3-VAR**		**P3-TET**		**P3-REL**	
P3-PAN			0.000		n.s.		0.000		n.s.	
	
P3-THC	0.000				0.000		n.s.		0.000	
	
P3-VAR	n.s.		0.000				0.000		n.s.	
	
P3-TET	0.000		n.s.		0.000				0.000	
	
P2-REL	n.s.		0.000		n.s.		0.000			
	

## Data Availability

No data reported.
